# Should I stay or should I go? The role of leadership and organisational context for hospital physicians’ intention to leave their current job

**DOI:** 10.1186/s12913-020-05285-4

**Published:** 2020-05-11

**Authors:** Pål E. Martinussen, Jon Magnussen, Karsten Vrangbæk, Jan C. Frich

**Affiliations:** 1 Department of Sociology and Political Science, Norwegian University of Science and Technology (NTNU), Trondheim, Norway; 2 Department of Public Health and Nursing, Norwegian University of Science and Technology (NTNU), Trondheim, Norway; 3 Department of Public Health / Department of Political Science, University of Copenhagen, Copenhagen, Denmark; 4 Institute of Health and Society, University of Oslo, Oslo, Norway

**Keywords:** Leadership, Personnel turnovers, Physicians, Organisation and administration, Clinical governance, Social environments, Norway

## Abstract

**Background:**

Physician turnover is a concern in many health care systems globally. A better understanding of physicians’ reasons for leaving their job may inform organisational policies to retain key personnel. The aim of this study was to investigate hospital physicians’ intention to leave their current job, and to investigate if such intentions are associated with how physicians assess their leaders and the organisational context.

**Methods:**

Data was derived from a survey of 971 physicians working in public hospitals in Norway in 2016. The data was analysed using descriptive statistics and multivariate analysis.

**Results:**

We found that 21.0% of all hospital physicians expressed an intention to leave their current job for another job. An additional 20.3% of physicians had not made up their mind whether to stay or leave. Physicians’ perceptions of their leaders and the organisational context influence their intention to leave their hospital. Respondents who perceived their leaders as professional-supportive had a significantly lower probability of reporting an intention to leave their job. The analysis suggests that organisational context, such as department mergers, weigh in on physicians’ considerations about leaving their current job. Social climate and commitment are important reasons why physician stay.

**Conclusions:**

A professional-supportive leadership style may have a positive influence on retention of physicians in public hospitals. Further research should investigate how retention of physicians is associated with performance related to organisational and leadership style.

## Background

Loosing well-performing physicians is costly for hospitals, and thus physician turnover is a critical issue in health care systems globally [1–4]. A better understanding of physicians’ reasons for leaving their job may inform organisational policies to retain key personnel. Previous studies have found that intention to leave a current job is associated with demographics, factors in the family or personal domain, working time and psychosocial conditions, job-related well-being and other career-related aspects [4, 5]. There is an increasing awareness of the role of organisational context for physician well-being, and factors that have been studied include the efficiency of the practice environment, the level of flexibility and autonomy, and expected workload [6–10]. Previous research has investigated relationships between retirement and organisationally related work stress, which can be modelled as perceptions of effect-reward balance and job strain [11]. It is likely that organisational factors influence physicians’ intention to leave their current job before retirement.

There is a gap in the literature on how leadership and the organisational context influence physicians’ intention to leave their current job. This may appear somewhat paradoxical, given the increasing focus on management and organisational change in healthcare. The wave of reforms across European health systems have led to a wide variety of changes in hospitals’ organisational structures. The reforms have been contested due to lack of evidence that the stated objectives have been achieved, ideological opposition to introducing market mechanisms in health care, and especially the reformers’ failure to consider health professionals’ opinions. It is therefore important to investigate how changes in leadership styles and new organisational structures are related to hospital physicians’ intention to leave their job.

The aim of this study was twofold: First, to investigate hospital physicians’ intention to leave their current job, and secondly, to investigate if such intentions are associated with how physicians assess their leaders and the organisational context. The relationship between leadership style and doctors’ turnover intent is of particular interest in a hospital setting, since leadership strategies and styles are something that can be changed within the organisation itself, as opposed to more external conditions related to institutional and environmental aspects. The study was based on a survey among Norwegian hospital doctors from 2016, where respondents rated the leadership qualities of their immediate leaders, as well as several items associated with current trends in the organisation and governance of hospitals. We first used factor analysis to identify two different leadership logics: ‘professional-supportive’ and ‘economic-operational’. We then used multivariate logistic regression to investigate whether turnover intent is related to leadership behavior and organisational context, controlling for relevant individual background variables such as gender, age, foreign medical exam, specialty and organisational climate.

### Institutional logics in healthcare

Health systems across the world have implemented reforms that call for a reconsideration of the role of management of hospitals, which is increasingly seen as important for performance. These efforts to reorganise hospitals have introduced new and more complex systems of management inspired by the business sector, gradually challenging and supplementing traditional profession-based management.

Many European health reforms during the last decades have been inspired by the doctrine of New Public Management (NPM), where the main idea is that market competition will improve service quality and deliver health services more efficiently. The reforms have thus strengthened patient rights and introduced choice, competition and financial incentives in a sector that has typically been state-directed and centrally controlled. In addition to business-like management principles the common elements have included the introduction of activity-based financing, purchaser-provider models, closer monitoring of performance, and performance-based contracts.

Efficiency concerns along with technological advances have led to hospital restructuring, introducing more flexible service delivery arrangements, seeking governance models that could centralise treatment functions, stimulate greater institutional autonomy and better integration across different types of services. Individual hospitals have therefore been given varying degrees of semi-autonomy within the public sector and have been empowered to make key strategic, financial, and clinical decisions themselves. Increased specialisation, a shift towards high-cost technological equipment and the necessity of having a sufficient patient volume in order to maintain quality for specialised services have typically resulted in decisions to reconfigure the hospital sector into fewer and larger administrative units [12].

Research on healthcare systems and reforms has shown that both managers and hospital physicians have had to adapt to these new institutional logics, following the increasing demands for efficiency and budgetary discipline [13–15]. The reforms have explicitly changed the conditions for management, e.g. by strengthening the role of management in relation to their political owners or external boards and emphasising market-like management practices. A central element of the reforms is to make clinicians better acknowledge the balance between clinical autonomy and managerial accountability, and to recognise how the clinical and financial dimensions of care are related [16]. The evolution in practice structure has thus created new challenges for physicians, requiring them to sacrifice some autonomy/flexibility, achieve productivity requirements set by the organisation, and be accountable to organisational leadership [17–21].

While few would question that “management matters” in delivering quality health care, knowledge about the nature of the relationship between management and performance is still incomplete [22]. In the literature, the importance of leadership is typically taken for granted, and the literature mainly discusses what constitutes a good leader rather than whether leadership makes a difference or not [23]. Critics of the concept of leadership claim that it has been too loosely defined to be useful, and that individuals – including leaders – account for very little variance in organisational performance [24].

It may be difficult to establish a clear causal relationship between organisational leadership and macro-level organisational factors such as financial performance, but leadership may make a significant difference at the individual and group levels of analysis [25]. Leaders engage in a range of behaviours that affect individual and team performance, and there is a long tradition of studies focusing on the role of leadership in healthcare for aspects such as quality of care, organisational commitment, job satisfaction, burnout and stress [23, 25–30]. Reflecting this, a review of leadership research in healthcare suggests that transformational leadership is associated with job satisfaction, extra effort, perceived unit performance, a supportive organisational climate, organisational commitment, and intention to stay and staff retention [25]. The review also identified consistent links between managers’ leadership style and staff job satisfaction, retention and organisational commitment. Notably, most of the participants in the reviewed studies were nurses, and the only health professionals other than nurses were social workers and mental health teams.

### The Norwegian health system

The Norwegian health care system is a tax-based systems and is similar to those of other Nordic countries, the United Kingdom, and Southern European countries such as Spain, Italy and Greece. Before the 2002 reform, the responsibility for primary care was devolved to the 435 municipalities, while responsibility for specialist health rested with the 19 counties. In this model hospitals were funded through global budgeting, with deficits having few practical consequences for management or physicians [31–33], and the hospitals enjoying little freedom to make decisions on internal organisation, investments, use of financial incentives, and other aspects.

While the Norwegian hospital system has been almost continuously under change during the last decades, there are in particular three reforms that have been informed by principles and models within NPM. First, in 1997 activity-based financing (ABF) replaced a proportion of the block grant with a matching grant based on diagnosis related groups (the ABF share has since 2013 remained stable at 50%). Second, in 2001 free choice of hospitals was introduced, allowing patients to choose other hospitals than their local hospital to avoid long waiting times. Third, with the hospital reform of 2002 the state took over the ownership of the county hospitals and all other specialised health services, organised the hospitals into health enterprises, and implemented private sector accounting systems. Common for all three reforms were their justification by cost increases and inability of hospitals to absorb patient inflow. Although not explicitly intended as management reforms, the result has been a wide variety of organisational changes in the hospitals: new divisions of tasks among hospitals, the introduction of incentive contracts, new and innovative technological solutions, new negotiating systems, benchmarking procedures, and changes in leadership and organisational cultures [33].

## Methods

The study was based on a survey from 2016 among hospital doctors in Norway. The hospital physicians were fully employed at a public hospital, although they may have had additional employment (usually no more than 20%) at private clinics. The hospital physicians have no regular panel of patients; they treat patients who were hospitalised or referred for outpatient consultations. The survey questionnaire was sent via Questback to a random sample of 3000 hospital physicians drawn from the register of medical practitioners, which at the time of the survey consisted of around 13,000 physicians in total. After four reminders, we ended up with 971 responders out of a gross sample of 2967, which gives a response rate of 32.7%. This may cause some concern, but it is still unclear whether a low response rate necessarily results in skewed samples and lower representativeness [34, 35]. Furthermore, we were able to assess the representativeness of our sample by comparing it with the members of the register of the Norwegian Medical Association, and found that the respondents in our data deviated marginally (only between 0 and 3%) from the register.

The survey asked respondents to rate the leadership qualities of their proximate leaders (department chair) on 11 specific dimensions on a 5-point Liker scare (1 = “does not emphasise”, 5 = “emphasises very much”), capturing the extent to which leaders are seen to prioritise various aspects of their job, such as promoting professional standards and quality in patient treatment, motivation of employees, solve interpersonal problems, ensure that rules and routines are followed, etc. (Table 1). The respondents also rated several items associated with current trends in the organisation and governance of hospitals, such as activity-based financing, absence of resident leadership, merging of departments into divisions/clinics and long lines of command. The dependent variable in this study was an item in the survey asking respondents the following question: “Are you currently having plans or wishes to leave this hospital in order to go to another workplace?”

**Table 1:** Factors, items loading, variance explained and internal consistence of the assessments of department leadership. Principal Component Analysis with Varimax Rotation with Kaiser Normalisation

Factors	1	2	Total
*Factor 1: Professional-supportive*			
Ensure professional standards and quality in patient treatment	.836		
Stimulate professional collaboration across different departments	.814		
Motivate employees and create support	.865		
Develop and utilize new routines and working methods	.768		
Coordinate different types of activity within the department	.743		
Solve interpersonal problems and differences	.802		
Initialize new professional opportunities	.853		
*Factor 2: Economic-operational*			
Economic steering, accounting and budget		.735	
Ensure that rules and routines are followed		.718	
% of variance	49.87	11.54	61.41
Cronbach alpha			.871

We first used factor analysis to identify two different leadership logics: “professional-supportive” and “economic-operational”. The former emphasises the promotion of medical standards and quality in patient treatment, while the latter focuses on economic management in terms of resource use and budgetary control, combined with practical organisational solutions and the management of the hospitals’ daily business. We then used multivariate logistic regression to investigate whether an intention to leave the current job was related to leadership behavior and organisational context, controlling for relevant individual background variables such as gender, age, foreign medical exam, specialty and organisational climate.

### Dimensions of leadership

Our data was found to be suitable for factor analysis through the KMO Measure of Sampling Adequacy, with a result of 0.89, and its significance was assessed through Bartlett’s test of Sphericity, with a result considerable lower than 0.05. The factor analysis was performed through Principal Component Analysis with Varimax Rotation with Kaiser Normalisation, and revealed the presence of two factors with eigenvalues greater than 1, capable of explaining 61.4% of the total variation (Table 1). The internal consistency of the leadership assessments as measured by the Cronbach alpha coefficient is .87, which indicates high interrelatedness between the items.

The first factor includes the tasks that can be associated with a professional logic: promoting professional standards and quality, stimulating professional collaboration, implementing new routines and working methods, and initializing new professional opportunities. In addition, this dimension also taps into the more supportive aspects of leadership, reflected in the high loading of the two items related to motivating employees and solving interpersonal problems. Hence, we choose to label this the “professional-supportive” leadership style. The second factor in Table 1 captures the more economic and operational factors embedded in hospital management. This logic is reflected in the two items that load high on the component of leadership here referred to as “economic-operational”. This type of leadership is founded on economic management, accounting, budgetary control, and adherence to formalised administrative routines and rules.

### Organisational context and social climate

The second set of explanatory variables in focus reflects the many organisational changes that have taken place in the hospitals the last decades. The survey asked respondents to score a number of organisational aspects/solutions on a 5-point Likert according to the degree to which they were seen as problematic with the current hospital model (1 = “unproblematic”, 5 = “very problematic”): a) absence of resident leadership, b) merging of departments into divisions/clinics, and c) long command lines.

Since turnover intentions can be expected to be related to organisational climate we also included two variables to reflect “social climate” and “commitment”. These two items were taken from The General Nordic questionnaire for psychological and social factors at work (QPSNordic), which has been thoroughly tested and tried in many organisations, and which is used by the National Institute of Occupational Health in Norway [36]. Social climate was measured through an additive index based on the following three statements about the social climate at the respondent’s work unit (5-point Likert scales as response format, where 1 = “very little/not at all”, 5 = “very much”): a) “encouraging and empowering”, b) “relaxed and agreeable”, and c) “rigid and rule-governed”. The engagement at the respondent’s working unit was captured through the following three statements (5-point Likert scales as response format, with 1 = “totally disagree”, 5 = “totally agree”): a) “I tell my friends that this is a good organisation to be employed in”, b) “My values mainly equals those of the organisation”, c) This organisation really inspires me to do my best”. The descriptive statistics for these variables are shown in Table 2.

**Table 2:** Descriptive statistics for the variables included in the analysis. Total sample size = 971. (0 = no, 1 = yes)

Variables		N
Professional-supportive leadership style	Mean: 2.87 Min.: 1 Max.: 5 St. dev.: .85	912
Economic-operational leadership style	Mean: 3.94 Min.: 1 Max.: 5 St. dev.: .65	932
Absence of resident leadership	0: 300 (31.8%) 1: 642 (68.2%)	942
Department mergers	0: 517 (54.5%) 1: 432 (45.5%)	949
Long lines of command	0: 240 (25.7% 1: 694 (74.3%)	934
Age < 40	0: 760 (80.0%) 1: 190 (20.0%)	950
Female	0: 567 (59.6%) 1: 385 (40.4%)	952
Foreign medical exam	0: 346 (36.4%) 1: 604 (63.6%)	961
Surgery (reference)	0: 589 (61.5%) 1: 368 (38.5%)	957
Internal medicine	0: 627 (65.5%) 1: 330 (34.5%)	957
Laboratory	0: 865 (90.4%) 1: 92 (9.6%)	957
Psychiatry	0: 823 (86.0%) 1: 134 (14.0%)	957
Other	0: 925 (96.7%) 1: 32 (3.3%)	957
Social climate	Mean: 3.05 Min.: 1 Max.: 5 St. dev.: .54	940
Engagement	Mean: 3.01 Min.: 1 Max.: 5 St. dev.: 1.14	947

For the purpose of the multivariate analysis the variable capturing physicians’ intention to leave their current job was recoded into a dummy-variable, with the value of 1 assigned to respondents with intention to leave the hospital. We analysed a subset of the data with the dependent variable coded as 1 for those who chose “yes” and 0 for those who chose “no” to the question of turnover intent, hence excluding the 193 respondents without an opinion. However, as this changed the results only marginally, they are not presented.

We controlled for possible confounding factors in our model. First, we included demographic background, captured through age and gender. A substantial share of physicians working in Norway have taken their medical exam abroad, and thus have their training from an organisational and institutional setting that may differ from the one they meet in Norwegian hospitals. Thus, “foreign exam” is also included as a possible confounding factor. Second, we also controlled for specialty by entering dummy-variables for internal medicine, laboratory, psychiatry and “other”, with surgery as the reference category. The descriptive statistics are presented in Table 2. Given that several of the independent variables in our model may be highly correlated, there is a potential concern for imprecise estimates due to large variance. However, collinearity diagnostics uncovered no such problems.

## Results

Out of 953 physicians who responded to the item “Are you currently having plans or wishes to leave this hospital in order to go to another workplace?”, 21.0% (*N* = 200) reported an intention to leave their current job, 58.8% (*N* = 560) responded “no”, while 20.3% (*N* = 193) reported no such plans or did not know. An interesting question is where those with an intention to leave their current job would like to work instead. In the survey, respondents replying “yes” were asked to report which type of working place or organisation they would prefer to go to. Table 1 shows that 21% expresses intention to leave public healthcare, with 28% preferring the private sector. The large majority of those preferring to stay in public healthcare favour going to another hospital (45%), with only 3% preferring municipal health services, and 8% other public health services (Table 3).

**Table 3:** Preferred working place for those with intention to leave their hospital (*N* = 200)

Preferred alternative working place	% (N)
Other hospital	44.5 (89)
Municipal health services / primary health care	3.0 (6)
Private healthcare	28.0 (56)
Other parts of public healthcare	8.0 (16)
Other type of work within healthcare	8.0 (16)
Don’t know/not applicable	8.5 (17)

Table 4 reports the results from the multivariate analysis. Given that the dependent variables are dichotomous, the model was estimated via logistic regression. The estimates in the table thus express the odds ratios for having quitting intentions. In order to uncover the role of the two sets of explanatory variables in focus, we first entered leadership style and organisational context in separate blocs, before including the controls in the final model. The models were estimated as fixed effects with dummy-variables for each of the hospitals in order to control for possible variation between hospitals not accounted for in the analyses.

**Table 4:** “Are you currently having plans or wishes to leave this hospital in order to go to another workplace?” (1 = “yes”, 0 = “no/don’t know”). Logistic regression with odds ratios, 95% CI in parenthesis. *N* = 857

	(1)	(2)	(3)
***Leadership style:***			
“Professional-supportive”	.32** (.25–.41)	.65** (.46–.91)	.64** (.46–.90)
“Economic-operational”	.97 (.74–1.27)	1.06 (.78–1.44)	1.04 (.76–1.43)
***Organisational context and social climate:***			
Absence of resident leadership		1.04 (.82–1.31)	1.06 (.83–1.34)
Department mergers		1.18 (.96–1.46)	1.20 (.97–1.50)
Long lines of command		.99 (.76–1.30)	.99 (.75–1.30)
Social climate		.52** (.33–.83)	.52** (.33–.82)
Commitment/value congruence		.51** (.39–.66)	.50** (.39–.66)
***Controls:***			
Age < 40			2.65** (1.73–4.28)
Female			.89 (.57–1.37)
Foreign medical exam			.77 (.50–1.21)
Internal medicine			1.03 (.64–1.67)
Laboratory			.60 (.26–2.29)
Psychiatry			1.22 (.65–2.29)
Other			1.67 (.37–7.48)
Constant	8.71**	52.16**	58.55**
Nagelkerke R^2^	.18	.30	.32

The relationships reported in the first column in Table 4 indicates that respondents who perceive their leaders to have a professional-supportive style have a significantly lower probability of turnover intent, with an odds ratio of .32 (CI = .25–.41). This relationship, however, increased to .65 (CI = .46–.91) when the set of variables reflecting organisational context enter the model (model 2). Including controls does not alter the relationship (model 3), with a reported odds ratio of .64 (CI = .46–.90).

Turning to organisational context, none of the three variables included are associated with intention to leave. Turnover intent is, however, associated with both the social climate and the engagement at one’s working unit: the more positive these aspects are evaluated, the lower the probability of reporting an intention to leave. The relationship is about the same for both variables: the odds ratio for social climate is .52 (CI = .33–.83) and .51 for engagement (CI = .39–.66).

The control variables were included in model 2, and young physicians have a higher likelihood of turnover intent, with a reported odds ratio of 2.65 (CI = 1.73–4.28). Gender, foreign exam or type of specialty was not associated with intention to leave.

Finally, we also estimated a model replacing hospital fixed-effects with dummy variables for regional background of the respondents, since many of the organisational aspects of the current hospital model are viewed as more problematic in the South-Eastern region of Norway, which was to be expected considering the wide-ranging reorganisation processes that have taken place and are still taking place in the region. However, the inclusion of dummy-variables for health regions did not alter the results.

## Discussion

The 2002 Norwegian hospital reform set the context for our analysis of the relationships between leadership, organisational features and physicians’ intention to leave their current job. The reform is indicative of the changes in formal organisation and leadership focus. The reform implied that the hospitals were turned into separate legal entities. Hence, even though ownership is still public, the hospitals are no longer an integral part of the central government administration. Central regulations are primarily to take place through the enterprise meetings, which correspond to the general assemblies in private enterprises. The introduction of an enterprise organisation signifies a distinct break from earlier administrative traditions, since it represents a new management philosophy: the enterprise structure implies an “arms-length” organisational division between the activity and the superior political body.

The Norwegian Hospital Act underlines that leadership should have the control and responsibility of all input factors, the authority to choose an organisational structure that advances the purpose of the activity, and complete responsibility for the management, without interference from other administrative levels. The responsibility and leadership dimensions are important in the reform: it strongly emphasises aspects such as distinct objectives, output demands and professional and genuine leadership, which are well known key words from the NPM doctrine.

Using physicians’ own assessments of their leaders and factor analysis, we were able to identify two different leadership styles. First, the response to the new demands of corporate and managed care are reflected in the leadership logic here referred to as economic-operational. This type of leadership is founded on economic management, accounting, budgetary control, and adherence to formalised administrative routines and rules. The other logic, termed the professional-supportive leadership style, emphasises the promotion of medical standards and quality in patient treatment, echoing both the historically strong role of medicine in the organisation and management of hospitals, as well as the promotion of new technologies and knowledge based on medical science.

Similar attempts of categorising healthcare professionals have been made by other researchers. First, the dimensions of leadership documented in our study bears some resemblance with a dichotomisation suggested by Degeling et al. [37]. Building on a survey among hospital staff, they distinguished between “clinical purists” and “financial realists” in the views on the clinical and resource dimensions of care. The former maintains that clinical judgement should be the sole basis for resource allocation, and that clinicians should not be accountable for their practice’s resource implications. The latter argues that clinical and resource issues are interrelated, since clinical decisions are necessarily also resource decisions. Johannesen and Olaisen [38] introduced a distinction between “enterprise professionals” and “health professionals” to describe the hospital actors in the wake of the Norwegian hospital reform. Here politicians and administrators belong to the first group and the rest are placed in the second category. The success criteria for the health professional perspective can be summed up as care, diagnosis, medication, quality and service, while the enterprise professional perspective builds on budgeting, accounting, economic management and market.

Using our distinction between leadership styles we found that the probability of physicians reporting an intention to leave their current job was one and a half times lower among respondents in departments characterized by professional-supportive leadership. At the same time we found that respondents’ intention to leave their current job was almost twice as high when the social climate was poor or commitment/value congruence was low. These two findings support our initial expectation that leadership and organisational context matters for physicians’ perception of their workplace and ultimately for their intention to leave.

Our findings may be regarded as further support to the observations by Gilmartin & D’Aunno [25], as our results also suggest that mergers influence physicians’ intention to leave their current job. This is an area of concern in light of research that shows that there is a trend in hospital management and organisation to establish fewer and larger administrative units [12, 25]. Given the debate in the Nordic countries it is relevant to note, that gender and specialty did not appear to matter for the results. Physicians’ age, on the other hand, seems to matter as physicians below 40 are more likely to report an intention to leave. This finding may to some extent be explained by the fact that younger physicians need to seek different residencies as part of qualifying to become a specialist doctor.

Turnover because physicians are not satisfied with their working conditions is costly for an organisation. Investments in building competencies are lost and replacement processes may be resource-demanding and time-consuming. When a specialised person is leaving an organisation services may be disrupted. Identifying organisation-specific predictors of turnover intent could lead to development of special recruitment and retention strategies for hospitals with high risk of losing physicians. Veth et al. [39] have suggested that relevant HR-strategies for retention of older employees in health organisations should include a combination of maintenance, development, utilisation and accommodative practices. In order to avoid a high rate of physician turnover, employers and managers may focus on creating and maintaining good working environments, developing an organisational culture and climate that builds trust and respects professional autonomy, while encouraging and recognising an individual’s performance [3, 4].

Results from a recent Danish survey among 2.736 senior physicians is in line with our findings [40]. Almost one-third of the senior doctors expressed a desire to retire before the age of 65. Younger doctors were more prone to indicate a desire for early retirement than older doctors. Four out of ten senior doctors pointed to dissatisfaction with working conditions as the main reason for wanting early retirement before the age of 70. In contrast to the Norwegian case it seems that specialty and region mattered for these results as doctors working in psychiatry and surgery and in the Danish Capital Region reported higher levels of dissatisfaction. About one out of three answered that they seldom or never receive recognition from management. A similar number reported a lack of respect and unfair treatment in the work place [40]. Although these data are not fully comparable, the tendency in a similar health care setting appears to corroborate our indications of the importance of organisational and leadership factors for physicians’ well being and intentions to leave the work place.

Limitations of our study include the low response rate and the single country case approach. Furthermore, we do not have data that allows a direct comparison with other professions, other countries or developments over time. Norwegian physicians are employed by the hospital, and it is possible that health systems with independent physician or physicians in a physician organisation may experience leadership and organisational factors differently. Repeating the survey over time could strengthen our results and provide indications of development trends in response to particular management policies. Another highly relevant way to understand our results in more detail would be to supplement qualitative studies about the specific reasons for intention to leave or to study effects of leadership styles in more in-depth [41, 42]. Finally, there are obviously several other factors in addition to leadership style and organisational context that may explain physicians’ leaving intentions, such as for instance working hours, frequency of night shifts, salary, patient violence, etc. Unfortunately, our data did not contain information that allowed us to investigate these aspects. Also, while intention to leave often reflects the level of frustration of the participants, it is unclear how many of them would eventually leave. However, the survey included no information about level of job satisfaction or other similar variables.

## Conclusion

We identified patterns in physicians’ intention to leave their current job within the hospital sector of Norway and observed that more than one fifth of all hospital physicians expressed intentions to leave their current organisations. An additional fifth were undecided. We further established that perceptions of leaders and organisational context matter for the intention to leave. Respondents who perceive their leaders to have a professional-supportive style have a significantly lower probability of intention to quit. Turning to organisational context, the analysis suggests that department mergers weigh in physicians’ considerations about leaving their current job. Other organisational factors did not have significant effects. However, both social climate and commitment are important for physician retention. A professional-supportive leadership style may have a positive influence on retention of physicians in public hospitals. A next step would be to expand the research interest to also include assessments of performance related to organisational and leadership style variables.

## Data Availability

The datasets used and/or analysed during the current study are available from the corresponding author on reasonable request.

## Abbreviations

ABF: Activity-based financing
NPM: New Public Management
QPSNordic: The General Nordic questionnaire for psychological and social factors at work
